# Design and Development of a Multi-Functional Bioinspired Soft Robotic Actuator via Additive Manufacturing

**DOI:** 10.3390/biomimetics7030105

**Published:** 2022-08-03

**Authors:** Nikolaos Kladovasilakis, Paschalis Sideridis, Dimitrios Tzetzis, Konstantinos Piliounis, Ioannis Kostavelis, Dimitrios Tzovaras

**Affiliations:** 1 Centre for Research and Technology Hellas—Information Technologies Institute (CERTH/ITI), 57001 Thessaloniki, Greece; n.kladovasilakis@ihu.edu.gr (N.K.); dimitrios.tzovaras@iti.gr (D.T.); 2 Digital Manufacturing and Materials Characterization Laboratory, School of Science and Technology, International Hellenic University, 57001 Thessaloniki, Greece; 3 RETOUCH-HS Private Company, 54636 Thessaloniki, Greece; sideridis@iti.gr (P.S.); cp@pnvalue.com (K.P.); gkostave@iti.gr (I.K.)

**Keywords:** bio-robotics, soft actuator, pneumatic motion system, additive manufacturing

## Abstract

The industrial revolution 4.0 has led to a burst in the development of robotic automation and platforms to increase productivity in the industrial and health domains. Hence, there is a necessity for the design and production of smart and multi-functional tools, which combine several cutting-edge technologies, including additive manufacturing and smart control systems. In the current article, a novel multi-functional biomimetic soft actuator with a pneumatic motion system was designed and fabricated by combining different additive manufacturing techniques. The developed actuator was bioinspired by the natural kinematics, namely the motion mechanism of worms, and was designed to imitate the movement of a human finger. Furthermore, due to its modular design and the ability to adapt the actuator’s external covers depending on the requested task, this actuator is suitable for a wide range of applications, from soft (i.e., fruit grasping) or industrial grippers to medical exoskeletons for patients with mobility difficulties and neurological disorders. In detail, the motion system operates with two pneumatic chambers bonded to each other and fabricated from silicone rubber compounds molded with additively manufactured dies made of polymers. Moreover, the pneumatic system offers multiple-degrees-of-freedom motion and it is capable of bending in the range of −180° to 180°. The overall pneumatic system is protected by external covers made of 3D printed components whose material could be changed from rigid polymer for industrial applications to thermoplastic elastomer for complete soft robotic applications. In addition, these 3D printed parts control the angular range of the actuator in order to avoid the reaching of extreme configurations. Finally, the bio-robotic actuator is electronically controlled by PID controllers and its real-time position is monitored by a one-axis soft flex sensor which is embedded in the actuator’s configuration.

## 1. Introduction

In the last decade, robotic systems and automation have undergone rapid development and implementation in various applications due to the increased need for automation arising from the fourth industrial revolution [1,2]. Thus, there is a demand to improve the existing robotic platforms and discover novel methods to produce and employ innovative robotic tools, such as grippers, novel mechanisms, etc., that will assist in agile production. The majority of robotic systems are fabricated with rigid materials, in order to handle increased mechanical loads, perform high precision motion and operate under demanding industrial environments [3]. However, due to safety reasons, the operation of these systems is limited in applications which require safe human–robot interaction. Hence, soft robotic systems draw increased interest for applications with real human–robot collaboration and robotic manipulations in tasks that involve fragile or sensitive objects, such as fruit grasping, etc. [4], which demand delicate handling.

The concept of soft robotics was bioinspired by the motion mechanisms of animals, such as worms, octopuses, human limbs, etc. [5], and this has led to the development of two types of soft robotic systems [6]. The first type utilizes soft elastomeric/rubber materials and external mechanical work produced by an electric motor. More specifically, the motor, with the assistance of a mechanical tendon, applies a force on the soft structure, which is deformed in the desired shape due to the material’s high elasticity [7,8]. The second type of soft robotics system performs continuous motions, employing smart materials or soft materials with smart structures, or soft materials integrated with a pneumatic system. In the first case, the structure is made of smart materials with shape memory characteristics and, under a specific stimulus such as temperature, electricity, pH, etc., develops internal stress resulting in external deformation of the structure in the desired shape [9,10]. The second case utilizes smart structures with the unique characteristic of a negative Poisson’s ratio, such as auxetic architected materials, in order to shift their external shape when an external force is applied [11,12,13]. The last case of a soft actuator employs a pneumatic system integrated with soft materials, which are the most widespread and studied soft actuators [14]. A plethora of developed soft pneumatic robotic systems has been presented in various scientific studies in the last decade [15,16,17,18]. For example, Wang et al. [15] and Udupa et.al. [16] developed positive-pressure pneumatic network actuators, also known as PneuNet actuators, which exploit their distinct design and positive pressure difference to achieve deformation. On the other hand, Brown et al. [17] presented a negative-pressure soft actuator capable of grasping objects by the application of a vacuum. Furthermore, other studies have employed complex concepts for pneumatic soft actuators, such as bellow-like structures [18], and concepts that utilize both types of soft actuators and integrate them into a single mechanism [6].

In the current study, a novel multi-functional soft actuator with a pneumatic motion system, bioinspired by the worm’s motion system, was designed, developed, and rapidly fabricated by combining different additive manufacturing (AM) techniques. The novelty of the developed actuator is summarized in the following three points:

- Maximum angular range from −180° to 180°, due to the bonding of two pneumatic chambers with mirror configuration.
- Multi-functionality and geometrical flexibility due to the modular configuration of the 3D printed external covers.
- Real-time monitoring of the actuator’s position due to the integration of the one-axis flex sensor in the actuator’s body.

**Figure 1:**
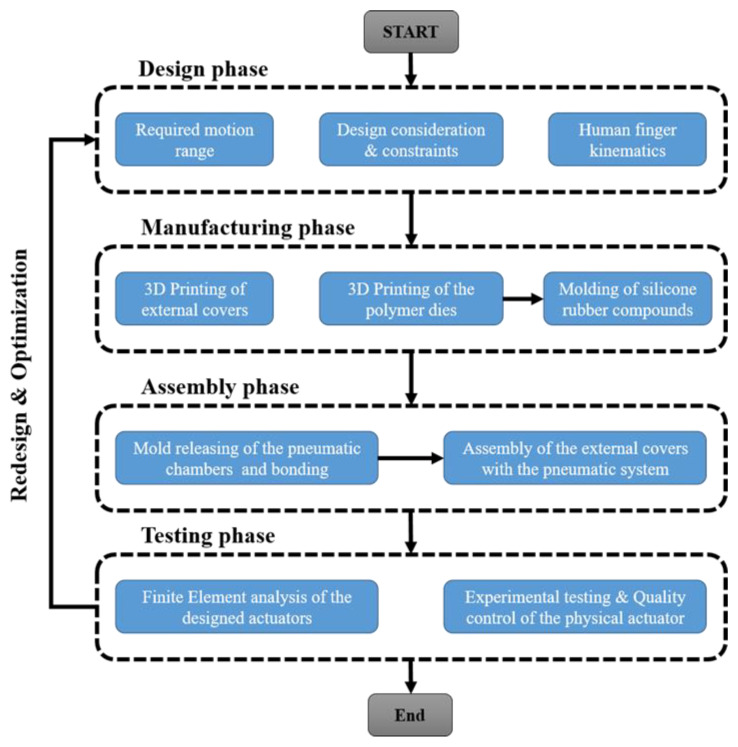
Flowchart for the production of the developed actuator.

Figure 1 illustrates the flowchart of the actuator’s production. More specifically, the actuator was designed to imitate the motion of the human finger with an enhanced angular range from −180° to 180°, taking into account the respective design considerations and constraints. An external modular cover was also designed in order to enhance the actuator’s structural integrity and multi-functionality. Then, the designed components were manufactured by employing additively manufactured dies for the molding of the pneumatic system and the selective laser sintering (SLS) AM technique for the external covers. The assembly process was performed with the assistance of Kevlar fiber reinforcement to improve the actuator’s durability. It is worth mentioning that the functionality, strength, and motion study of the designs were evaluated through finite element analyses (FEA), and the final physical actuators were tested via quality, endurance, and mechanical tests. Finally, potential applications of the developed soft actuator were examined and analyzed.

## 2. Materials and Methods

### 2.1. Design Phase of Soft Robotic Actuator

The first step towards the design of soft actuators comprises the establishment of some practical requirements and basic constraints to use them as guidelines for the manufacturing process. The first requirement concerns the weight and the volume of the actuator. According to the existing literature [19,20], the weight of a soft robotic actuator that imitates the human finger should not exceed 0.1 kg and its volume and dimensions should be comparable with an actual human finger of an adult man [21]. Thus, the dimensions of the actuator should be lower than 200 mm in length (measured from the center of the palm), 25 mm in width, and 35 mm in height. The next step is the observation of a human finger motion, in order to establish the minimum angular range of the actuator. Human fingers have three degrees of freedom (DOF), apart from the thumb, which has two DOFs, resulting in a sum of 250° and 160° degrees of inwards rotation, respectively [19,22,23]. In addition, the motion of hyperextension of a finger could reach up to 30° degrees (external rotation) [19,22]. Furthermore, the human finger generates a grasping force of approximately 7.3 N [24,25], so this value is the minimum mechanical requirement for the actuator’s grasping force. In addition, the actuator should have a similar motion speed to the human finger and be able to function for multiple open–close cycles in a row.

After the presentation of the design requirements and constraints, the next process is the design of the multi-functional soft actuator. The design of the pneumatic chambers imitates the structure and the locomotion of an earthworm. According to studies of the existing literature [26,27,28], earthworms, such as Lumbricus Terrestris, employ peristaltic and undulatory locomotion in order to move in a 3D space. In detail, these motions occur when specific groups of worm muscles are expanded or contracted, in a way that produces bending elongations along the earthworm’s body. In order to imitate this locomotion, the designed structure should be able to expand a specific region of the actuator much more than the others. This could be achieved with a half-tubular construction with a rigid linear side pressurized internally by a working fluid, in this case, air, leading to a significant expansion of the half-tubular side, which results in the bending elongation of the structure.

**Figure 2:**
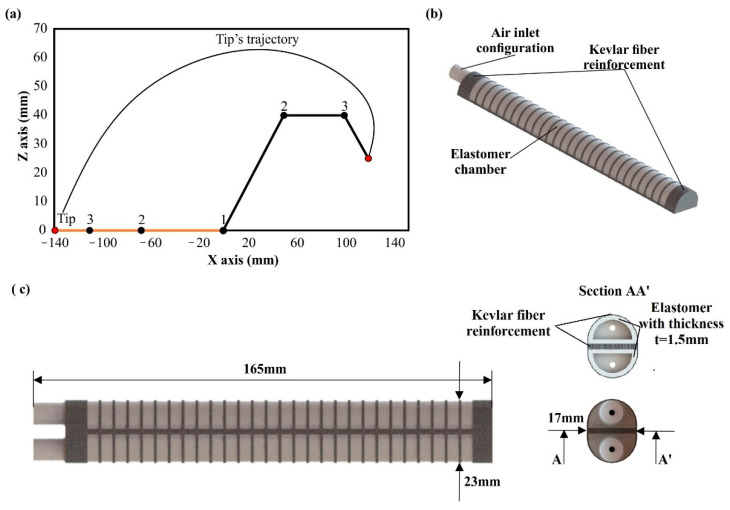
(**a**) Indicative 2D diagram of a human finger motion; (**b**) Half-tubular construction of soft actuator; (**c**) 3D models of the overall soft actuator with dimensions.

The dimensions and the shape of this chamber are illustrated in Figure 2 as it was designed in the SolidWorks™ software. More specifically, the dimensions of each chamber were 165 mm × 17 mm × 11 mm for the length, width, and height, respectively. However, one half-tubular air-pressurized chamber has a limited angular range for extension motion. Thus, it was decided to bond two half-tubular air-pressurized chambers with their flat surface, as it is portrayed in Figure 2. This configuration allows the maximum angular range (−180° to 180°) and increases the generated force for both bending and extension motions. The bonding of the chambers was performed at the two tips of each chamber in order to achieve minimum shear stresses on the actuator’s body. Moreover, an inlet configuration was designed on each chamber in order to connect the chamber with an air-pressurized pneumatic system. It is worth mentioning that a silicone rubber compound was selected as the construction material. This type of material has high elasticity and handles increased elastic deformation (above 100% of strain) before their yielding point. However, its ultimate mechanical strength is limited when a force or pressure is constantly applied and could lead to plastic deformation and failure of the silicone rubber compound. Hence, it is essential for the pneumatic chamber’s structure to be reinforced with an external structure to achieve uniform deformation of the silicon rubber structure without regions of intense stress concentration [29,30,31]. The reinforcement of the pneumatic chamber was performed by applying the Kevlar fiber around the elastomeric structure. It is worth mentioning that different applications of Kevlar fiber were tested in order to find the optimum combination and achieve the maximum durability of the structure [32,33].

### 2.2. Manufacturing and Assembly of Soft Actuator

**Figure 3:**
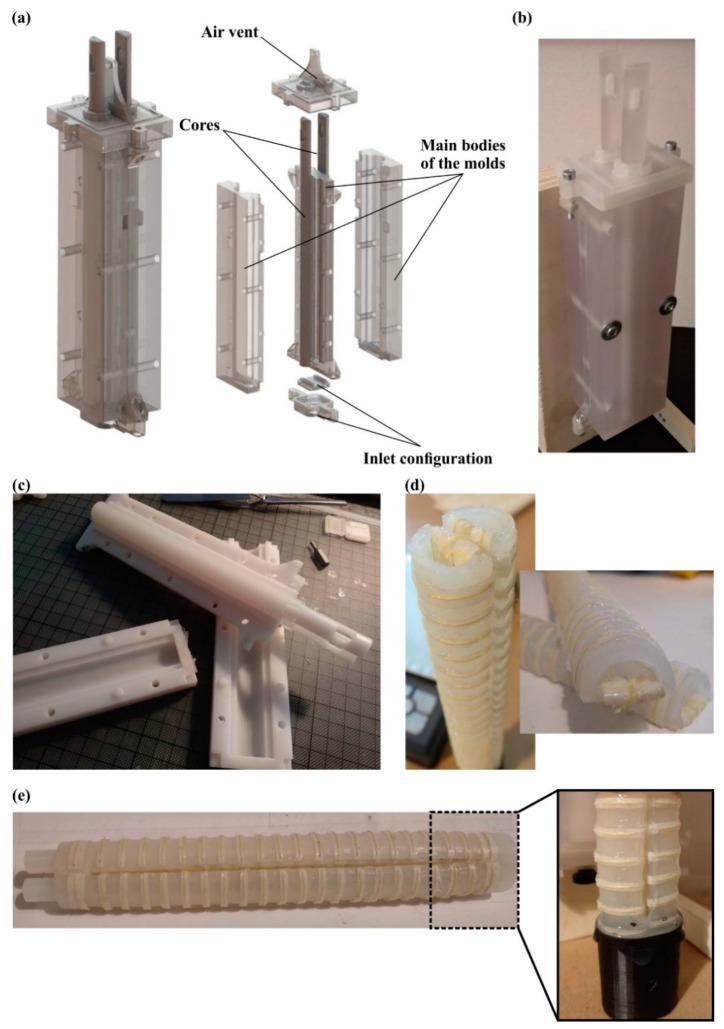
(**a**) A 3D model and exploded view of the silicone molds; (**b**) Image of the 3D printed mold; (**c**) Releasing process of the pneumatic chamber from the molds; (**d**) Indicative Kevlar reinforcement of the pneumatic chambers; (**e**) Bonding and final design of the soft actuator.

The manufacturing process of the soft actuator is divided into two stages: the fabrication of the pneumatic chambers and the additive manufacturing of the external covers. As has been mentioned above, a silicone rubber compound was selected as a construction material for the pneumatic chambers due to its high elasticity and sufficient strength. In order to produce high-fidelity parts, a silicone molding procedure was employed utilizing additive manufacturing molds/dies. More specifically, the design of the dies was extracted from the 3D models of the pneumatic chambers and was amplified with air vents, for the extraction of the air during the molding process, and sealing parts to ensure that the molds were leakproof, as it is depicted in Figure 3a,b. The material jetting AM technology was employed, namely the ProJet^®^ MJP 5600 (3D Systems, Rock Hill, SC, USA) 3D printer, for the production of the molds, due to its high dimensional accuracy up to 25 μm (16 μm layer height) [34]. In detail, for the external parts of the mold, a rigid polycarbonate-like transparent material, i.e., the VisiJet^®^ CR-CL 200 (3D Systems, Rock Hill, SC, USA), was utilized to monitor the flow of the silicone inside the mold. Furthermore, the cores of the mold were manufactured with a white ABS-like material, i.e., VisiJet^®^ CR-WT 200 (3D Systems, Rock Hill, SC, USA), to facilitate the monitoring of the flow of silicone. The 3D printing and post-processing of the molds were rapid procedures as they were performed in almost 5 h (3 h and 30 min for the printing and 1 h and 30 min for the post-processing). Furthermore, the Dragon Skin™ 20 (Smooth-On Inc., Macungie, PA, USA), which is formulated from two compounds of silicone rubber, was chosen as the construction material for the pneumatic chambers due to its high strength and low cure time. Table 1 lists the basic physical and mechanical properties of the Dragon Skin™ 20 and the VisiJet^®^ materials according to the manufacturers. The mixing of the silicone compounds occurred by utilizing static mixers, and the molding process was performed in a vacuum chamber to avoid air bubbles inside the silicone rubber mixture. After the molding process, the produced pneumatic chambers were released from the mold, as it is shown in Figure 3c. The molding process was conducted in less than 5 h, with 30 min for the mixing, injection, and de-bubbling procedures, and 4 h for the curing of the silicone rubber material.

The next step comprised the reinforcement of the silicone parts with Kevlar fiber (Figure 3d). In the context of the current study, three different methods of Kevlar fiber reinforcement were developed and tested. The first method concerned the wrapping of the Kevlar fiber around the silicon rubber chamber with a constant pitch. The second method concerned the embedding of the Kevlar fiber inside the structure of the pneumatic chamber during the molding process. The last method was the sewing of the Kevlar fiber on a fabric saturated with silicone and then bonding it to the pneumatic chamber with a silicone rubber adhesive, i.e., Sil-Poxy^®^ (Smooth-On Inc., Macungie, PA, USA). These methods underwent quality control tests of open–close cycles, and the method with the best performance was applied in the final design of the soft actuator, as it is described in Section 3.2. The last step of this stage comprises the bonding of two pneumatic chambers with the proper orientation via the silicone molding process, as it is presented in Figure 3e.

The final fabrication stage is the additive manufacturing of the external covers. The external covers provide structural integrity to the soft actuator’s structure and offer multi-functionality on the device due to their modular structure and the ability to change their material depending on the task. More specifically, in this study, two different materials were employed based on the desired application. The first material was a rigid polymer, namely the Polyamide 12-PA12 (Sinterit, Poland), for a hand exoskeleton application, and the second material was a thermoplastic elastomer, namely the Thermoplastic Polyurethane Flexa™-TPU (Sinterit, Poland), for a soft gripper application. Both versions of the external covers were fabricated with the selective laser sintering (SLS) AM technique via a Lisa Sinterit (Sinterit, Poland) 3D printer, due to the advanced print-out mechanical properties and accuracy. Table 2 summarizes the main parameters of the SLS 3D printing process. The SLS 3D printing process was the most time-consuming step, i.e., 20 h of 3D printing time; however, it is an independent procedure that could be conducted in parallel with the other manufacturing steps, such as the molds’ 3D printing, the molding process, etc.

**Figure 4:**
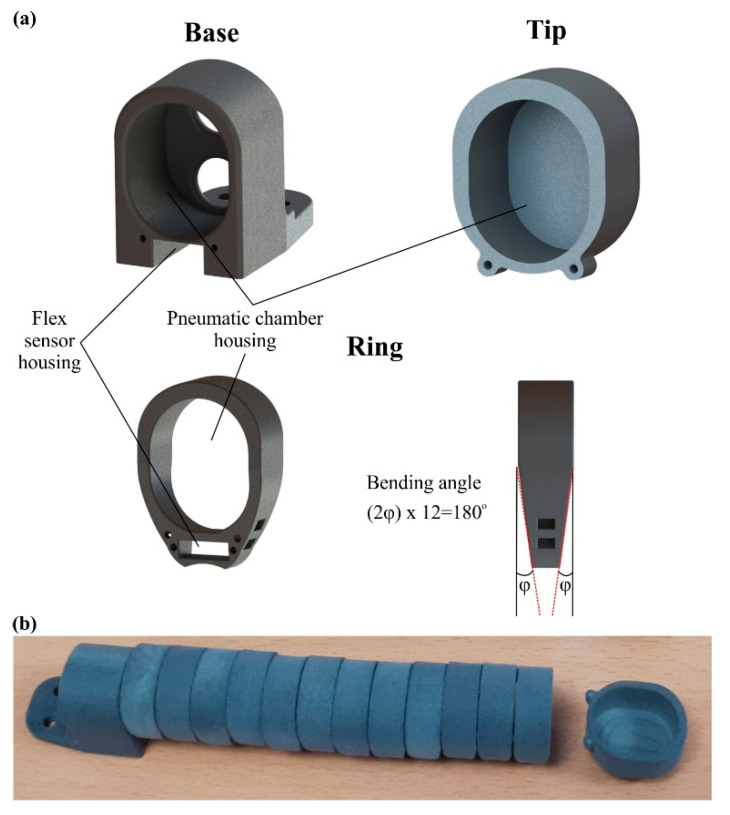
(**a**) 3D models of the base, ring, and tip; (**b**) 3D printed external covers made of PA12.

The external covers were designed in a modular manner consisting of one base, twelve identical rings, and one tip in the row, as it is shown in Figure 4. These parts have a central hole that fits with the soft actuator structure and a rectangular opening for the one-axis soft flex sensor, tailored to monitor the state of the actuator in real-time. Furthermore, the rings possess a unique geometry with specific chamfers of the structure that determine the maximum angular range for the open and close motion, in order to avoid undesired angles (Figure 4a).

### 2.3. Quality Control, Finite Element Analyses and Testing

**Figure 5:**
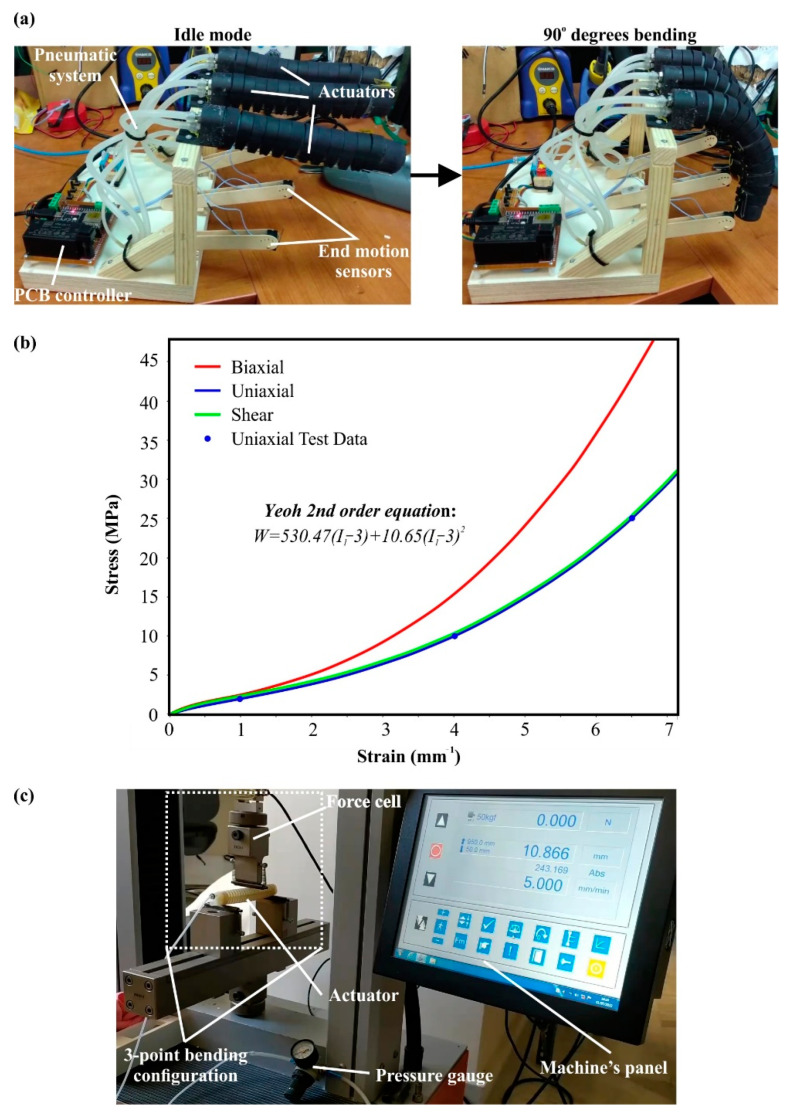
(**a**) Setup of the quality control process; (**b**) Yeoh 2nd order stress–strain curves for the hyper-elastic material model; (**c**) Experimental layout for the deflecting force measurements.

Each produced soft actuator underwent a series of quality control tests in order to verify its reliability and functionality. Firstly, after the molding process, the pneumatic chambers were visually inspected for discontinuity within the silicone rubber mass, such as air bubbles and impurities. The pneumatic chambers with visible flaws were disqualified for the next stages of the manufacturing process. Before the assembly of external covers on the soft actuator’s body, the fabricated actuator was tested for its functionality and durability through consecutive and iterative open–close cycles with an angular range from −180° to 180°. Then, after the assembly of the external covers, the same process was repeated to secure the integrity and the correct kinematic motion of the final structure. It is worth mentioning that at least 50 consecutive open–close cycles occurred for each quality test process. Moreover, the optimum method of Kevlar reinforcement was selected via the same quality control setup until the catastrophic failure of the actuator due to the high number of consecutive open–close cycles. Figure 5a presents the setup of the quality control process.

One of the most crucial steps in the manufacturing of such products is the finite element analysis (FEA) of the item, in order to simulate the operating conditions and extract the main physical and mechanical properties, such as stresses, strain, deformations, etc. For this purpose, the ANSYS™ software platform (ANSYS, Inc., Canonsburg, PA, USA) was employed with its static analysis module. In the context of the current study, FEA was developed for one pneumatic chamber due to the symmetric structure of the soft actuator and the limited element numbers. Hence, the analysis simulated the motion of a pneumatic chamber with Kevlar and cloth reinforcement. The computational mesh of the analysis consisted of 58,983 tetrahedral elements with an element size of around 1.3 mm. The aforementioned mesh emerged after the conduction of mesh sensitivity analyses in order to achieve mesh-independent results. It is worth mentioning that the elastic response of the actuator was utilized for the mesh sensitivity analysis [35]. Furthermore, fixed supports were employed on the structure around the air inlet configuration, and 200 kPa pressure was applied on the internal surfaces of the pneumatic chamber as a loading condition. In addition, 50 sub-steps were needed in order to smoothly converge the solving algorithm and capture the large deformation of the structure. Finally, the finite element analysis utilized two different material models to simulate the mechanical response of reinforcement and the mechanical behavior of the silicon rubber chamber. For the reinforcement structure, an isotropic material was employed with a density of 1.44 g/cm^3^, elastic modulus at 4900 MPa, and Poisson’s ratio at 0.36; these values are similar to Kevlar’s properties (according to the manufacturer) and slightly increased to capture the contribution of cloth on the reinforcement structure. On the other hand, the material model of silicone rubber was more difficult to be simulated; thus, a hyper-elastic material model was utilized. More specifically, the hyper-elastic model 2nd order Yeoh was employed by curve-fitting it with the material properties of Dragon™ Skin 20, which are listed in Table 1. The 2nd order Yeoh model utilizes the formulation described in Equation (1), with the strain energy density (W) and the first strain invariant (I_1_), along with incompressibility factors (d_1_, d_2_), volume ration (J), and materials constants (C_1_, C_2_), to extract the desired hyper-elastic mechanical response [36,37,38]. In this analysis, the material was assumed as incompressible, hence J = 1, and material constants were evaluated after the curve-fitting process at 530.47 kPa for C_1_ and 10.65 kPa for C_2_. Figure 5b shows the Yeoh 2nd order diagrams that were applied for the developed finite element model.

(1) $$W=C_{1}\left(I_{1}-3\right)+C_{2}{\left(I_{1}-3\right)}^{2}+\frac{1}{d_{1}}{\left(J-1\right)}^{2}+\frac{1}{d_{2}}{\left(J-1\right)}^{4}=C_{1}\left(I_{1}-3\right)+C_{2}{\left(I_{1}-3\right)}^{2}$$

The testing phase of the designed soft actuator was performed via a series of measurements and experiments. The first experiments concerned the connection between the applied air pressure inside the chamber with the bending angle of the soft actuator, measured with a protractor and verified with the one-axis flex sensor during the open–close cycle. Through this process, the kinematic of the actuator was observed and, depending on the applied pressure, was curve-fitted with the 3rd order polynomial function in order to be able to predict the position of the soft actuator based on the applied pressure or to calculate the necessary pressure to achieve the desired bending angle. The second round of tests was focused on the produced deflecting force of the soft actuator. The calculation of the deflecting force occurred via a three-point bending configuration. In detail, the actuator was positioned on two fixed points on the two edges of the actuator, and the force was measured in the middle of the actuator where the maximum force is expected, as it is shown in Figure 5c. For the purpose of this experiment, the universal testing machine Testometric-M500-50AT (Testometric Company Ltd., Rochdale, UK), equipped with a 500 N load cell, was utilized. Moreover, the acquired experimental data of the deflecting force and air pressure were curve-fitted with a 3rd order polynomial function in order to create a formulation that links the applied air pressure with the produced deflecting force. In addition, it is worth mentioning that the applied air pressure was monitored with the assistance of a pressure gauge positioned in the inlet of the pneumatic chamber.

## 3. Results and Discussion

### 3.1. Final Design of the Soft Actuator

**Figure 6:**
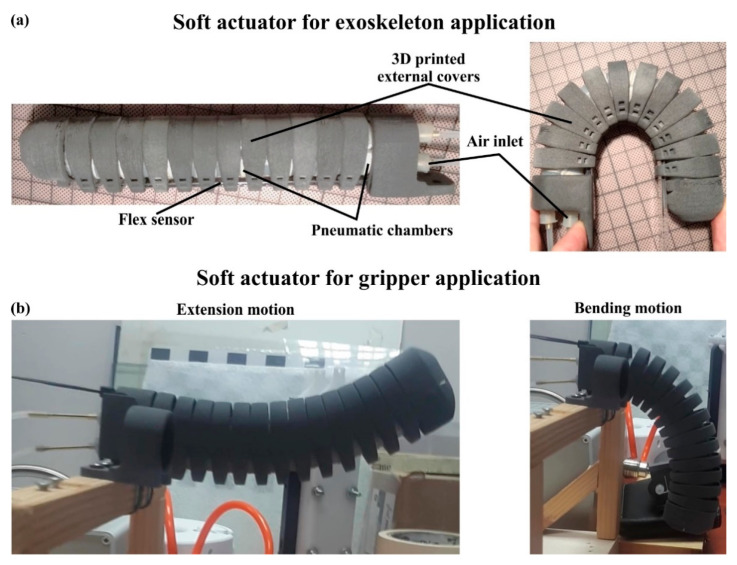
The designed actuator in maximum extension and in maximum bending position for two applications: (**a**) An exoskeleton application; (**b**) Gripper application.

In this subsection, the produced soft actuator is presented along with the assembly of external covers and the one-axis soft flex sensor. After the manufacturing and bonding of the pneumatic chambers, the next step was the assembly of them with the 3D printed external covers. The pneumatic chambers were assembled with the base in the corresponding openings for the chambers and the air inlets. In this step, a silicone rubber adhesive was applied between the base and the chamber to secure the assembly. Then, the rings were consecutively positioned covering almost the whole chambers, i.e., twelve rings, except from a surface of 18 mm. This uncovered surface was used for the assembly of the tip (15 mm), which was performed in a similar way to the base. The remaining surface of 3 mm was intentionally left uncovered in order to provide small gaps between the 3D printed parts and avoid their friction during the operation. The next step was tightening up the assembly with Kevlar fibers via the small holes on all 3D printed parts (Figure 4a). In that way, the structural integrity of the actuator was enhanced and the structure’s degrees of freedom were limited, prohibiting the rolling of the external rings over the chamber. Finally, the one-axis flex sensor was positioned through corresponding openings in the 3D printed parts and bonded with the base, utilizing a mild adhesive to secure the correct positioning. It is worth noting that the whole manufacturing and assembly process of the actuator consist of rapid production procedures, as it could be conducted in less than a day (≈22 h); in addition, with the proper equipment, a large number of actuators could be fabricated simultaneously. Figure 6 portrays the overall assembly of the designed actuator in the maximum extension and maximum bending position for two different applications/functions, namely for an exoskeleton application (Figure 6a) and gripper application (Figure 6b).

To control the developed actuator, the one-axis flex sensor and special miniature solenoid valves were employed in a specific configuration and driven by a software controller. In particular, a combination of three three-way valves was utilized in order to drive or exhaust the two pneumatic chambers of the actuator, resulting in an active closing and opening movement. The employed controller was a PID (proportional integral derivative) that receives, as control feedback, the difference (error) between the current position by the flex sensor and the set goal angle of the actuator. The output of the controller, due to the binary state of the valves (on–off), is the percentage of time that the valve is turned on for a defined time period. After tuning the PID coefficients through trial-and-error testing, a smooth and accurate movement was achieved without overshoot and offset errors.

### 3.2. Quality Control, Finite Element Analyses and Testing

All versions of the designed soft actuator were examined via the quality control process that was presented in Section 2.3. More specifically, the major concern of the soft actuator was the durability during the open–close cycles and the pressure variations. Thus, the final design should be able to perform a sufficient number of open–close cycles without structural failure. During this investigation, it was observed that the Kevlar reinforcement around the silicone rubber pneumatic chamber possesses a crucial role in the actuator’s durability. Hence, three different methods of Kevlar reinforcement were applied and tested, as was mentioned in Section 2.2. The method with the lowest performance in terms of open–close cycles was the one that embedded the Kevlar fiber inside the structure of the pneumatic chamber during the molding process, with around 1250 open–close cycles. This occurred due to the fact that the molding of the Kevlar inside the silicone rubber matrix introduced flaws in the interface regions between the two materials leading to a lower mechanical performance. The method of wrapping the Kevlar fiber around the silicon rubber material with a constant pitch revealed slightly higher durability, with almost 2850 open–close cycles, because of the lack of discontinuity inside the silicone rubber material. However, the major defect of this method was that the small diameter of the Kevlar fiber tore the silicone rubber material during the application of high pressures, which resulted in the failure of the actuator. The best performance in terms of durability appeared from the method that sewed the Kevlar fiber on a fabric saturated with silicone and then bonded it to the pneumatic chamber with a silicone rubber adhesive, with close to 14,800 open–close cycles. In this method, the developed stresses were distributed uniformly on the surface of the actuator leading to a significantly higher number of open–close cycles due to the existence of a fabric saturated with silicone between the Kevlar fiber and the silicon rubber material. Table 3 lists the numbers of total open–close cycles. Moreover, the following FE analysis shows the stress concentration on the Kevlar reinforcement due to the bending of the actuator.

**Figure 7:**
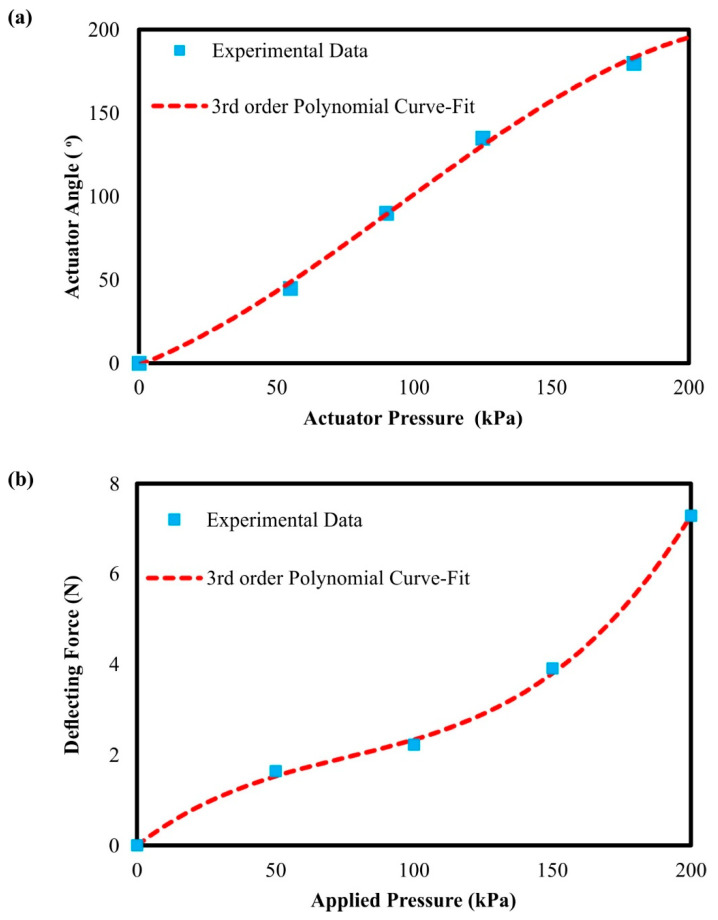
Graphical relationship between: (**a**) the applied pressure and the bending angle of the actuator; and (**b**) the deflecting force produced by the actuator depending on the applied pressure.

After the quality control process and the determination of the best method for the actuator reinforcement, the next step of the study was to perform a series of experiments and measurements in order to evaluate the actuator’s performance and gather the necessary data for the calibration of the FE model. First, the bending angle was connected with the applied pressure, utilizing a protractor and a pressure gauge. The actuator’s bending angle is defined by the angle of the arc that is formed between the first (idle position—0° degrees) and the final position of the actuator’s tip. It is worth mentioning that the measurements were verified by exploiting the measurements of the one-axis flex sensor. Figure 7a depicts the measurements for an angular range from 0° to 180°, coupled with the graphical relation between the applied pressure and the bending angle. For variations in the low air pressure region (<100 kPa), the changes in the bending angle are small with an almost linear relationship; as the actuator moves into higher bending angles, it requires higher changes in pressure resulting in a flattening of the curve. Thus, a third order polynomial equation is required in order to accurately capture the relationship between the applied pressure and the bending angle, which is presented in Equation (2). It is worth noting that Φ is the actuator’s bending angle, p_in_ is the applied air pressure, and polynomial coefficients were calculated by curve-fitting the curve on the experimental data. The next measurement was focused on the calculation of the deflecting force produced by the actuator during its operation (Figure 7b). According to the acquired data, for low applied pressures below the 50 kPa, the relationship between the produced force and the applied pressure was close to linear. For moderate applied pressures between 50 kPa and 100 kPa, the slope of the curve was severely reduced. However, after 100 kPa applied pressure, the produced force increases at an exponential rate. Hence, a high applied pressure range (>100 kPa) was required in order to achieve sufficient deflecting forces. After multiple repetitions of the experiments, the maximum deflecting force, that secured the actuator’s structural integrity, was measured at 7.298 N. Furthermore, a numeral third order polynomial function was built, exploiting the acquired data via the curve-fitting process (Equation (3)), where F_d_ is the deflecting force and p_in_ is the applied pressure.

(2) $$\Phi =-4.15\times 10^{-5}\times p_{\mathrm{in}}^{3}+0.011\times p_{\mathrm{in}}^{2}+0.327\times p_{\mathrm{in}}$$

(3) $$F_{d}=1.83\times 10^{-6}\times p_{\mathrm{in}}^{3}-4.19\times 10^{-4}\times p_{\mathrm{in}}^{2}+0.047\times p_{\mathrm{in}}$$

**Figure 8:**
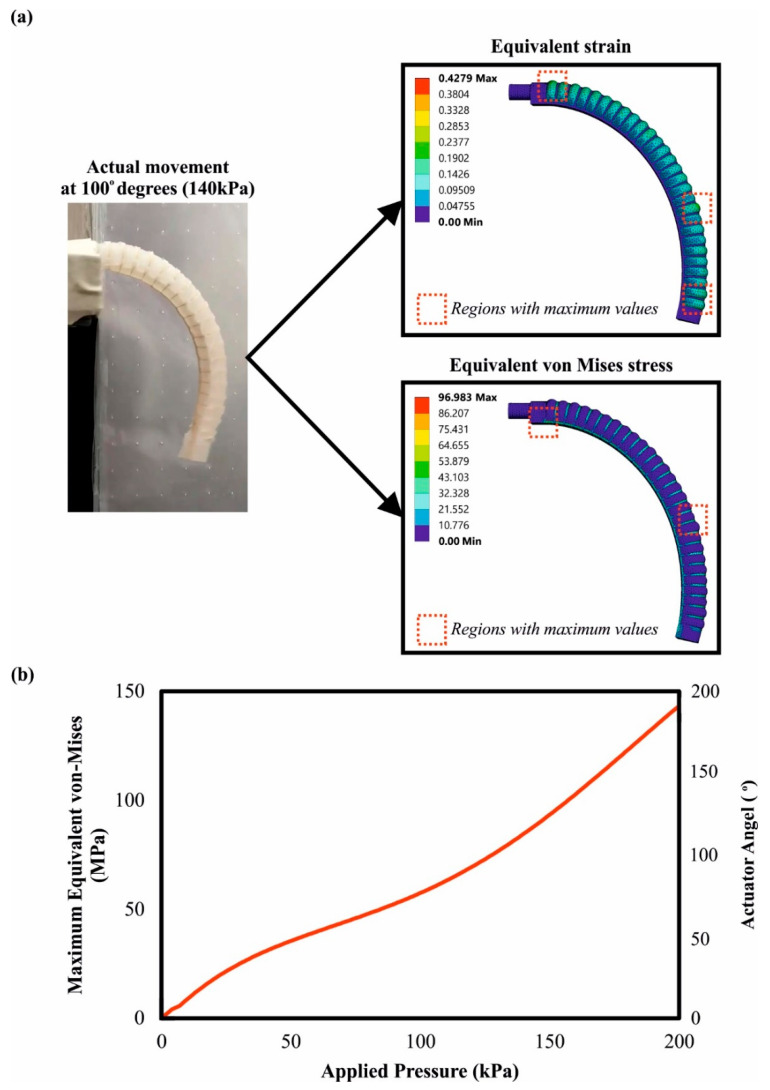
(**a**) Indicative images of the actuator’s motion derived from the physical testing and the FEA; (**b**) Graphical relations between the applied pressure, the bending angle, and the developed von Mises stress on the actuator.

Exploiting the experimental data, a finite element analysis was performed in order to accurately simulate the movement of the pneumatic chamber and observe the regions of stress concentration during the operation. The motion simulation facilitated the integration of the device into robotic systems and allowed the prediction of the position and kinematic of the actuator depending on the input parameters (air pressure). On the other hand, stress concentration contours highlighted the regions that withstood the highest loads in order to improve them in the next design iteration. In Section 2.3, the developed finite element model was described and analyzed. Moreover, the solving algorithm converged after almost 51 min and 225 iterations. Figure 8a illustrates a snapshot of the actuator bending from 0° to 100°, coupled with superimposed images of the regions with the maximum equivalent von Mises stresses. As was expected, there were two regions with the highest stress concentration; the first region was located at the bottom region near the fixed support, and the second was at the Kevlar fiber in the middle of the top side of the actuator. Both of these stresses were caused by the tensile loads that were applied in the regions due to the bending of the actuator and the inflation of the actuator. Furthermore, Figure 8b shows a diagram with three axes that concern the FEA results and connects the applied pressure with the developed von Mises stresses and the bending angle of the actuator. Moreover, acquired experimental data are also presented in this chart in order to verify the accuracy of FEA analysis. At the maximum bending angle and pressure, the maximum stress occurred with a value of 140 MPa on the Kevlar structure, which is below the Kevlar tensile strength. In addition, the stress to the applied pressure curve followed a third polynomial trend, with a slow increment of the stress in the beginning (low pressure, small angle) and a rapid increment with an exponential rate after 90° due to the high applied pressure (>100 kPa) and bending angle. It is noteworthy to point out that, during the motion, the silicone rubber component experienced a mild deformation with a maximum value of strain close to 70%.

### 3.3. Potential Applications

**Figure 9:**
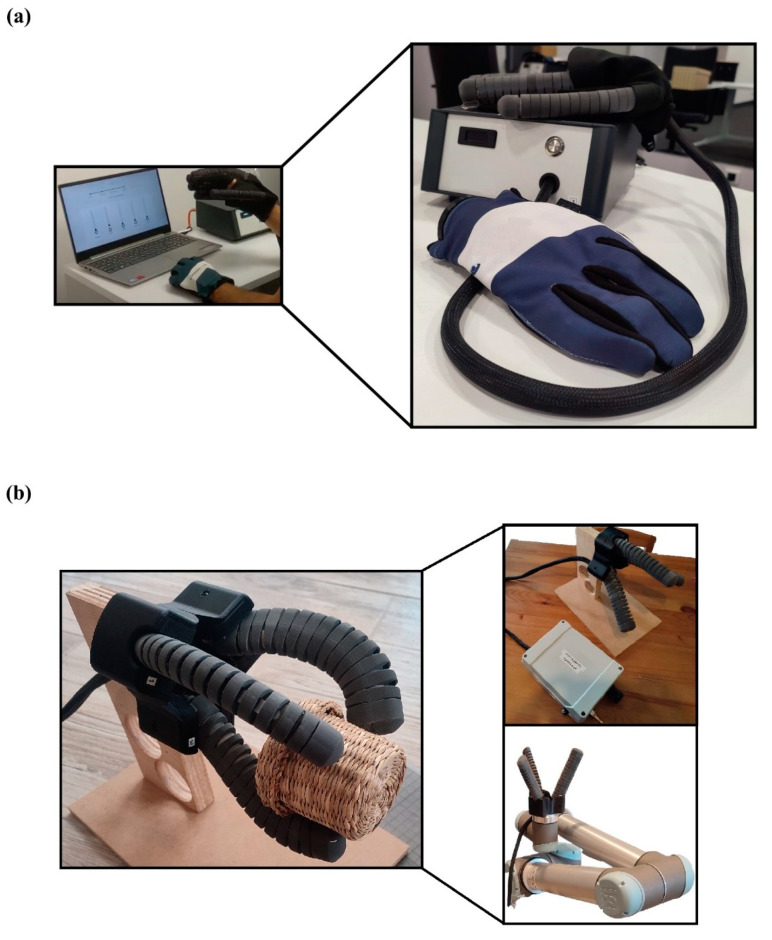
Indicative applications for the developed actuator: (**a**) Exoskeleton for medical applications to assist patients with mobility difficulties; (**b**) Soft gripper for the handling of delicate objects.

In the current study, a soft actuator was developed and produced with multiple functionality purposes. In detail, in the context of this research, the developed soft actuator was combined with others and tested in two different realistic-use cases. It is worth mentioning that the external 3D printed covers were altered in terms of material and geometry for each case depending on the requirements and constraints of the application. The first case was to assemble five actuators on a glove to constitute a human hand exoskeleton, according to guidelines in the literature [39,40]. Thus, each actuator was bonded to the external side of each finger of the glove, as it is presented in Figure 9a. These hand exoskeletons could be utilized by patients with mobility difficulties and neurological disorders. In this case, the exoskeleton has two applications; the first is for rehabilitation purposes in order to restore the functionality of the damaged limb, or for patients that have completely lost the functionality of their finger. For this application, the external covers were made of rigid polymer (PA12) and the angular range was selected from 0° to 180°, without enabling the extension motion for negative degrees.

For the second case, three soft actuators were assembled with a 3D printed bracket in order to produce a soft gripper compatible with a series of Universal Robots 10, i.e., UR10, as it is presented in Figure 9b. The uses of this soft gripper include the grasp, pick up and placement of fragile objects, such as fruits (grapes), without damaging their external surface or structure, through delicate grasping. The developed device had a three-jaw gripper configuration with each finger positioned in circular patterns with 60° spacing. For this case, the 3D printed external covers were fabricated from elastomer material (Flexa™-TPU) and the angular range was chosen between −30° and 90°, due to the fact that the extension motion is essential for grasping/picking functions. It should be stressed that the design of the actuators’ mounting base was modified in order to tightly assemble with the 3D printed bracket. In future scientific research, other potential uses of the developed actuator could be investigated and tested in various commercial applications.

## 4. Conclusions

In the current paper, the overall production process of a multi-functional bioinspired soft actuator was presented from its design to the manufacturing phase, and two types of application have been demonstrated. More specifically, the developed actuator was conceptualized and designed by imitating the worm’s motion mechanism. Furthermore, the actuator was fabricated with a silicone molding process via 3D printed polymer molds/dies. Then, the optimum method for the reinforcement of the actuator with Kevlar fiber was investigated and applied. In addition, 3D printed external covers were designed and additively manufactured in order to improve and secure the structural integrity of the actuator. The developed modular design of the external covers provided the multi-functionality of the device, as the covers’ material and geometry could be modified depending on the desired application. The overall production processes could be performed in a short time period, i.e., less than a day. Moreover, a series of tests were conducted on the produced actuators in order to extract the necessary numerical tools for the function of the actuator. Hence, the deflecting force and the bending angle could be estimated based on the applied pressure on the inlet of the actuator. In addition, through these tests, the maximum deflecting force was evaluated at 7.298 N for 200 kPa pressure, and the durability of the actuator was measured at 14,752 open–close movement cycles. In order to enhance the motion study of the produced actuator, a finite element analysis was performed and this accurately simulated the motion and kinematics of the actuators. It should be mentioned that the regions of stress concentration and the deformation of the actuator were observed via FEA, with a maximum stress value of almost 140 MPa and a maximum strain of close to 70%. Finally, two different uses of the developed actuators, namely a hand exoskeleton and a soft gripper, were presented, and the necessary design modifications were analyzed. Future research could focus on the strength of the actuator’s in order to increase the operation pressure, and achieve higher forces at the tip and enhanced durability. Furthermore, a scaling down of the actuator would be an interesting study that could be conducted to enable microelectromechanical (MEMS) applications.

## Figures and Tables

**Table 1:** Physical and mechanical properties of the Dragon Skin™ 20 the VisiJet^®^ materials.

Material	VisiJet^®^ CR-WT/CR-NT 200	Dragon Skin™ 20
Density (g/cm^3^)	1.16	1.08
Cure time (h)	-	4
Shore A/D hardness	77–80D	20A
Tensile strength (MPa)	33–43	3.79
Tensile modulus (MPa)	1400–2100	0.34
Elongation at break (%)	12–22	620
Tear resistance (kN/m)	-	21.02

**Table 2:** Main parameters of SLS 3D printing process for the external covers.

Parameters	Flexa™-TPU	PA12
Powder’s PSD ^1^	D_50_ = 33.5 μm and D_90_ = 39.5 μm	
Layer height	75 μm	
Laser power	5 W	
Scan speed	100 mm/s	
Beam diameter	0.4 mm	
Chamber temperature	160 °C	177.5 °C

^1^ PSD: Particle size distribution, measured with scanning electron microscope.

**Table 3:** Quality control results for the three reinforcement methods.

Methods	Kevlar Fiber Wrapped with Constant Pitch	Kevlar Fiber Embedded in The Elastomer Matrix	Kevlar and Silicone Fabric Bonded on The Actuator
Motions	5692	2494	29,504
Cycles	2846	1247	14,752

## Data Availability

Not applicable.
